# A meta-synthesis of studies on ubuntu philosophy in nursing: Implications for nursing education

**DOI:** 10.4102/curationis.v48i1.2652

**Published:** 2025-02-28

**Authors:** Vhothusa E. Matahela, Nelisiwe Ngwenya

**Affiliations:** 1 Department of Health Studies, College of Human Sciences, University of South Africa, Pretoria, South Africa; 2 Department of Quality Assurance, KwaZulu-Natal College of Nursing, Pietermaritzburg, South Africa

**Keywords:** African philosophy, holistic nursing, humanness, integration, meta-ethnography, meta-synthesis, nursing education, patient-centred care, ubuntu

## Abstract

**Background:**

Ubuntu, an ancient African philosophy, embodies the essence of humanness through communal responsibility and interconnectedness. It shapes moral values and culture that can be implemented in nursing to improve patient care. However, colonial and Western-centric education systems have marginalised ubuntu philosophy resulting in a disconnect from its core principles in contemporary nursing practices.

**Objectives:**

This meta-synthesis aims to synthesise qualitative literature on ubuntu philosophy in nursing to identify key aspects of the philosophy that can be integrated into nursing education.

**Method:**

A systematic search using keywords related to ubuntu in nursing and nursing education was conducted across multiple databases, including health sources: Nursing/Academic Edition, Medline, ProQuest, PubMed, CINAHL and Google Scholar. This search aimed to identify qualitative studies exploring the application of ubuntu philosophy within nursing and its implications for nursing education. Quality appraisals were conducted using the Critical Appraisal Skills Programme checklist for qualitative research. Noblit and Hare’s meta-ethnography techniques were employed to synthesise studies.

**Results:**

A total of 16 studies were included. The meta-synthesis revealed one meta-theme, namely the integration of ubuntu principles into nursing education with three subthemes: pedagogical approaches, decolonising nursing education and role modelling ubuntu.

**Conclusion:**

Ubuntu should be integrated into formal nursing education programmes to align with patients’ expectations and enhance nursing care quality and student needs for targeted support on the subject.

**Contribution:**

The study enriches the ongoing discourse on integrating indigenous philosophies such as ubuntu into nursing education, aligning nursing care with local cultural and ethical values to promote more humane practices.

## Introduction

Ubuntu, described as the essence of humanness, is a deeply rooted tradition embedded in tribal practices across southern Africa. Although a difficult concept to translate or express in English, ubuntu is defined by Mangaliso (2001:21) as ‘humanness, a pervasive spirit of caring and community, harmony and hospitality, respect and responsiveness that individuals and groups display for one another’. As a way of life, ubuntu recurs as maxim across the various African languages in southern Africa: in Zulu ‘Umuntu ngumuntu ngabantu’ or ‘Motho ke motho ka batho ba bang’ (Sotho) and in TshiVenda ‘Muthu ndi muthu nga vhanwe vhathu’ meaning that ‘a person is a person through other persons’.

Ubuntu involves communal responsibility and a deep interconnectedness, reflecting a unique indigenous perspective on moral values and culture. Ubuntu is an abstract concept that is supported and concretised by some elements, such as respect for persons, community, personhood and morality. Ubuntu fosters human excellence, as one’s life improves when one embodies this philosophy (Metz 2019). This philosophy also cultivates a sense of mutuality that reduces individual ego and tendencies to harm the community. This intrinsic aspect of ubuntu distinguishes humanity from other beings. Moreover, ubuntu involves identifying with others, which means seeing oneself as part of a collective ‘we’ that shares pride or shame in collective activities and cooperates in joint endeavours to achieve common or compatible goals (Metz 2019).

The ubuntu philosophy is deeply ingrained in caring science, holding a revered position within the nursing field in South Africa (Mulaudzi et al. 2022; Nolte & Downing 2019). It values subjective experiences and their significance in individuals’ lives, serving as a foundation for holistic and personalised care in nursing (Tembo 2023). However, African youth have deviated from their indigenous beliefs, and this deviation has persisted even decades after political liberation, which is attributed to colonial education systems that prioritise Western worldviews (Ngubane & Makua 2021). Consequently, this disconnect erodes ancestral wisdom, leaving the younger generation detached from their cultural heritage and sense of identity (Ngubane & Makua 2021). The colonial and apartheid political systems have institutionalised societal racism, leading to a segregated education system, and curricula that did not challenge apartheid ideologies. Consequently, ubuntu values, which traditionally upheld the preservation of cultural identity and communal bonds, were conspicuously disregarded and weakened.

Matahela and Van Rensburg (2023) observe that as with other sectors, the apartheid system also shaped the structure of the country’s nursing education and training, reflecting and reinforcing values of racial division and oppression where cultural diversity was systematically excluded. Nurses were divided and trained according to their racial backgrounds. Black nursing students, in particular, were primarily trained to care for their own communities, but the curriculum was rooted in a biomedical model that neither addressed their specific cultural needs nor incorporated African traditional customs and ways of life (Beal 1970; Pentecost et al. 2018). This approach led to the persisting disparities in access, quality and inclusivity that affect the offering of nursing education and training (National Department of Health 2020).

Contemporary nursing practices have faced criticism for their dehumanising nature, characterised by a decline in respect, empathy and integrity within the practitioner–patient relationship (Bvumbwe & Mtshali 2018). An example is when nurses sometimes dehumanise patients by referring to them not by name, but by their diagnosis or bed number (Chabeli 2001). Such attitudes and environments can create distressing experiences for patients, who may feel dehumanised and treated as mere objects rather than individuals. These kinds of behaviours hinder the facilitation of reflective thinking among nursing students, as they do not model respect for human rights and compassion.

The loss of the human aspect in care is often attributed to the dominance of the biomedical model, which views the human body as a mechanical object, devoid of historical, cultural or social context (Gordon 1988). This perspective reduces the complexity of the human experience, placing a higher priority on physical ailments and technological interventions than on holistic patient care. Even in post-colonial South Africa, the biomedical paradigm remains prevalent, perpetuating a Eurocentric approach to patient care that often overlooks the cultural and personal dimensions of health (Joyner, Shefer & Smit 2014; Pentecost et al. 2018). Widespread reports reveal instances of unprofessional and unethical conduct within nurse-patient relationships, marked by unethical conduct, poor communication, and occurrences of violence and abuse (National Department of Health 2013). Additionally, the caring behaviours of certain graduate and registered nurses continue to be identified as inadequate and inappropriate (Muhammad-Lawalet al. 2023a).

It is concerning that nurse educators, entrusted with the responsibility of facilitating teaching and learning in nursing education institutions, often overlook the ubuntu philosophy, a doctrine that is deeply rooted in African culture and capable of shaping perceptions of care. Often nurse educators involved in the teaching and learning of students prefer and emphasise Western caring concepts and theories (Muhammad-Lawal et al. 2023b). This preference can be attributed to some educators perceiving indigenous philosophies as inferior to those from other parts of the world. Additionally, there is a lack of motivation among some educators to write, discuss, defend, lecture or tutor on the subject (Mugumbate et al. 2023).

The scarcity of educational resources that incorporate ubuntu philosophy further complicates its integration into academia. McGibbon et al. (2014) highlight a significant gap in post-colonial knowledge within South African nursing literature. Educators often find themselves without access to syllabi, textbooks, journal articles, case studies, codes of ethics, guidelines, models, theories and various digital content that reflect ubuntu values (Mugumbate et al. 2023). Students typically learn about ubuntu through informal social interactions in their communities (Mulaudzi, Libster & Phiri 2009; Rasweswe et al. 2023), creating a disconnect between formal nursing education and patients’ expectations of care (Mulaudzi et al. 2022).

As a well-defined philosophy and a way of life in numerous African communities, ubuntu has garnered a substantial body of empirical and theoretical research. This philosophy has been tested and applied across various professions and disciplines including nursing (Shambare 2021). Despite its rich foundation in humanness and communal responsibility, the ubuntu philosophy is marginalised in South Africa’s formal nursing education system. Therefore, a vital element of African moral values and culture is notably missing in the training and professional development of nurses. Although the ubuntu philosophy is deeply rooted in caring ethics in South Africa, there is a growing concern that nurses may be straying from the time-honoured philosophy of ubuntu. There are also observations that suggest that nurses, especially the younger generation, lack the motivation to embody ubuntu’s principles, missing out on the moral guidance it provides (Mulaudzi et al. 2022). There is a substantial body of research on ubuntu in nursing, yet few identify key aspects of this philosophy that could be integrated into nursing education. Nurse educators often overlook ubuntu philosophy in favour of Western caring theories and concepts, potentially rendering nursing education irrelevant to the South African population’s health needs (Muhammad-Lawal 2023a).

The gap between formal nursing education and patients’ care expectations underscores the importance of transforming learning and teaching through integrating ubuntu principles (Muhammad-Lawal et al. 2023a). A detailed study that synthesises ubuntu in nursing and provides insights on incorporating ubuntu principles into nursing education could empower nurse educators to integrate ubuntu’s core values into their teaching-learning methods. This, in turn, would foster a nursing practice that honours both the autonomy of individual nurses and the communal spirit inherent in ubuntu. Evidence suggests that care deeply rooted in a patient’s cultural and moral framework leads to improved health outcomes (Younas, Inayat & Masih 2023). Currently, there is a noticeable disconnect between the formal training nurses receive and the care expectations of patients and communities (Zulu 2023). Thus, exploring ubuntu through a meta-synthesis can reveal methods to make nursing care more personalised and culturally relevant to the population by influencing how nurses are trained.

A preliminary literature search was conducted to gain insights into the topic before undertaking the meta-synthesis. The population, intervention, comparison, outcomes and study design (PICOS) framework (Methley et al. 2014) was applied to formulate the research question guiding this meta-synthesis. In this study, the population to which the review findings apply comprises student nurses. The intervention involves integrating ubuntu philosophy and its principles into nursing education, with the comparison focussing on approaches in nursing education that do not incorporate ubuntu. The anticipated outcomes include insights that support the preparation of nursing students for the complexities of a 21st-century health system. The study design includes qualitative studies that focussed on ubuntu in nursing and its educational implications. The emerging research question was:

What insights from qualitative literature on ubuntu philosophy in nursing can be integrated into nursing education to prepare students for the dynamic health system of the 21st century? (authors research question)

In response to this question, the study, through its objective, aims to conduct a meta-synthesis that reviews existing literature from qualitative studies on ubuntu philosophy in nursing to identify the key insights and aspects of ubuntu that should be integrated into nursing education.

## Research methods and design

### Study approach and design

This study employed a meta-synthesis of qualitative data through meta-ethnography, adhering to a constructivist-interpretative paradigm as described by Creswell and Creswell (2023). Noblit and Hare’s (1988) meta-ethnographic approach guided the meta-synthesis. This approach is a highly utilised and impactful methodology for qualitative evidence synthesis in health and social care research (Flemming & Noyes 2021). The researchers preferred a meta-synthesis method because it synthesises a collective body of qualitative research to identify common themes and/or to compare and contrast them on a general topic/concept, providing deeper insights that might not be available in a single study with its own context (Gray& Grove 2021). This endeavour resulted in a greater contribution to understanding how ubuntu philosophy could be inculcated into nursing education after reviewing and synthesising the selected studies, culminating into a greater applicability (Lachal et al. 2017).

The meta-synthesis approach is comprised of seven phases that overlap and repeat as the meta-synthesis progresses. In the first phase, getting started, the researchers defined the study’s research question and scope. In the second phase, we decided what would be relevant to the initial interest to establish criteria for selecting studies based on our research question and theoretical framework, justifying our inclusion and exclusion decisions, and ensuring transparency and coherence. In the third phase, we read the studies, engaging in deep and repeated readings of the selected studies, immersing ourselves in their data, interpretations and findings. We also took detailed notes and created excerpts to capture key concepts. In the fourth phase, we determined how the studies are related, focussing on identifying thematic connections and relationships between the selected studies. Here we further explored similarities and differences, considering factors like context, methodology and findings. The fifth phase involved translating the studies into one another. This meant rigorously analysing, and translating concepts and findings from one study into the language and framework of another, thus allowing us to build bridges between studies and identify higher-order constructs. The sixth phase, synthesising translations, involved bringing together the translated findings from different studies to develop a broader, synthesised understanding of the research question. We identified patterns, discrepancies and emergent themes, creating a richer understanding of the topic. The last phase, expressing the synthesis, involved communicating our findings concisely. Thus, this article will attempt to present the research question, methodology, analysis and synthesised understanding of the aspects related to integrating ubuntu philosophies in nursing education. Where applicable, we created diagrams, tables or other visual representations of our findings.

### Search strategy

The population of this meta-synthesis were qualitative research studies that explore ubuntu philosophy in nursing, with implications for nursing education. The studies had to be published in the English language in any given year, without any chronological restrictions to ensure a comprehensive scope. Relevant keywords encompassing ubuntu and nursing education formed the core of the search strategy. These keywords, drawn from specific categories, were amalgamated to establish search terms in the following format: (ubuntu-related terms) AND (nursing education-related terms), employing truncation where necessary. Keywords and phrases related to ubuntu and nursing education formed the core of a search strategy including ‘ubuntu philosophy’, ‘Nursing education’, ‘ubuntu values in nursing’, ‘African philosophy in nursing education’, ‘Integrating ubuntu in nursing curriculum’, ‘Communal values in nursing education’, ‘ubuntu ethics in healthcare education’, ‘African perspectives in nursing training’ and ‘Socialisation of nursing students with ubuntu’. These terms/phrases were used in combination using Boolean operators (AND, OR) to refine and target search results effectively.

Both researchers independently searched for literature between December 2023 and May 2024. The search involved searching studies from the inception of database publications *without* any chronological restrictions to ensure a comprehensive scope. The university librarian was consulted to assist with a comprehensive review of electronic databases, thus reducing bias. Multiple databases, including Health Source: Nursing/Academic Edition, Medline, ProQuest, PubMed and CINAHL were thoroughly explored. Additionally, Google Scholar was utilised to survey grey literature, employing search phrases like ‘ubuntu in nursing education’ and ‘individualism and collectivism in nursing education’ to retrieve studies, with the inclusion of the first 100 studies from each query. Furthermore, an exploration of the reference lists of included studies was conducted to identify supplementary relevant literature.

### Study selection

The two researchers screened titles and abstracts against the inclusion criteria. Full-text articles that appeared relevant were then retrieved and independently assessed by the researchers for inclusion based on the identified criteria. Decisions on whether to include an article were reached through consensus.

Inclusion criteria covered qualitative studies such as ethnography, phenomenology, grounded theory, narrative analysis and case studies focussed on ubuntu in nursing and its educational implications. Mixed method studies were included if they clearly stated the methodology, but only the qualitative components were used for this meta-synthesis (Nolte et al. 2017; Yang et al. 2023). In terms of the type of study, eligible sources included original research, peer-reviewed articles, book chapters, dissertations, theses and conference proceedings. These types of studies were chosen for their potential to provide rich qualitative data.

Exclusion criteria encompassed quantitative studies, randomised controlled trials, systematic reviews and studies that do not directly address ubuntu philosophy within nursing education. In addition, studies that were not original research, such as non-peer-reviewed articles, editorials, opinion pieces or non-academic publications, were excluded to maintain academic rigour.

### Quality appraisal

#### Critical appraisal

The quality appraisal was accomplished through the utilisation of the Critical Appraisal Skills Programme (CASP) (2018) tool for qualitative studies. The CASP tool is recognised as the most commonly used tool for quality appraisal in health-related qualitative evidence syntheses (Long, French & Brooks 2020). Comprising 10 questions, this tool evaluates the quality and validity of qualitative research by scrutinising the appropriateness of methods, presentation and meaningfulness of findings. Both researchers independently evaluated and scored the quality of each study. The scores were averaged, and any discrepancies were resolved through discussion until consensus was achieved. We then compared each other’s results, discussing any discrepancies to reach a consensus. Despite the absence of specific grading guidelines or cut-off points for inclusion in the CASP tool, a score was calculated by assigning one point to each question, resulting in a total score of 10. Importantly, we did not exclude studies based on quality appraisal as the aim was to identify aspects of ubuntu philosophy that can be integrated into nursing education. Nevertheless, the CASP tool played a pivotal role in contributing to the Confidence in the Evidence from Reviews of Qualitative research (CERQual) approach for assessing confidence in review findings (Lewin et 2018). Confidence in the Evidence from Reviews of Qualitative research determined confidence levels based on methodological concerns, relevance, coherence and adequacy of studies (Lewin et 2018). High confidence scores were assigned to studies with minor methodological issues and high coherence, relevance and adequacy. Conversely, studies with major limitations or low coherence/relevance received low confidence. Studies with mixed scores were allocated moderate confidence levels. The confidence scores served the purpose of identifying studies with a reasonable representation of the phenomenon of interest, that is, key aspects or insights of ubuntu philosophy that can be integrated into nursing education (Lewin et al. 2018).

#### Rigour

To ensure rigour in this meta-synthesis, we adopted a comprehensive and multi-layered approach throughout the research process, integrating diverse empirical and theoretical sources. The study was conducted in distinct stages to uphold methodological integrity and minimise bias. A well-defined and systematic literature search strategy was developed and executed across multiple databases, with the assistance of the university librarian. This ensured that our search was not only computer-based but also incorporated diverse approaches, including ancestry searching, journal hand-searching, networking and searches of research registries. These strategies were employed to capture both published and grey literature, ensuring a thorough representation of the available evidence. Therefore, the use of multiple data sources enhanced methodological rigour, thereby integrating different viewpoints and ensuring a more comprehensive and nuanced understanding of the topic, strengthening the credibility of the findings.

To maintain transparency and consistency, specific inclusion and exclusion criteria were established prior to the literature search. These criteria guided the selection process, ensuring that only relevant and high-quality studies were included. To avoid selection bias, both the researcher and an independent reviewer independently screened the studies against these criteria. Any discrepancies were resolved through discussion, and there was no need for an independent reviewer to be consulted. The data extraction process was conducted independently by both the researcher and an independent reviewer. Each reviewed the selected studies to ensure that key information was consistently and accurately extracted. In instances where there were disagreements, an independent reviewer assisted in reaching a consensus, further enhancing the reliability of the findings. This rigorous process minimised the risk of bias and ensured that all perspectives were considered. To assess the quality of the included studies, we employed an established critical appraisal tool, namely the CASP. We also engaged in ongoing reflexivity to remain aware of their own positionality and potential biases that might influence the synthesis. This reflective practice helped mitigate any preconceptions and maintained the objectivity of the analysis.

### Data extraction and synthesis

Given the qualitative nature of the review, the planned method for synthesising data involved a meta-ethnographic approach as outlined by Noblit and Hare (1988). This approach entails a systematic and iterative process of comparing and synthesising qualitative research findings. Initially, key concepts, that have implications for ubuntu philosophy’s integration into nursing education, were identified across selected studies. Then, a line of argument synthesis was employed to understand the relationships and interactions between these concepts. This synthesis involved a process of constant comparison, coding, and categorisation of findings from the included qualitative studies. To explore these concepts further, the researchers employed thematic analysis to identify recurrent themes or metaphors that emerge regarding the integration of ubuntu principles in nursing education. This process involved a careful examination of data, allowing for the identification of patterns and themes that encapsulate the essence of ubuntu’s incorporation into nursing education. The researchers organised, coded and analysed the qualitative data manually.

### Ethical considerations

Ethical clearance to conduct this study was obtained from the University of South Africa, College of Human Sciences Research Ethics Review Committee (Reference no.: 90553624_CREC_CHS_2024). The study was also registered with PROSPERO register of systematic reviews (reference no.: CRD42024497462). The review adhered to ethical principles at every stage, including conceptualisation, planning, implementation and dissemination.

## Results

### Included studies

**FIGURE 1:**
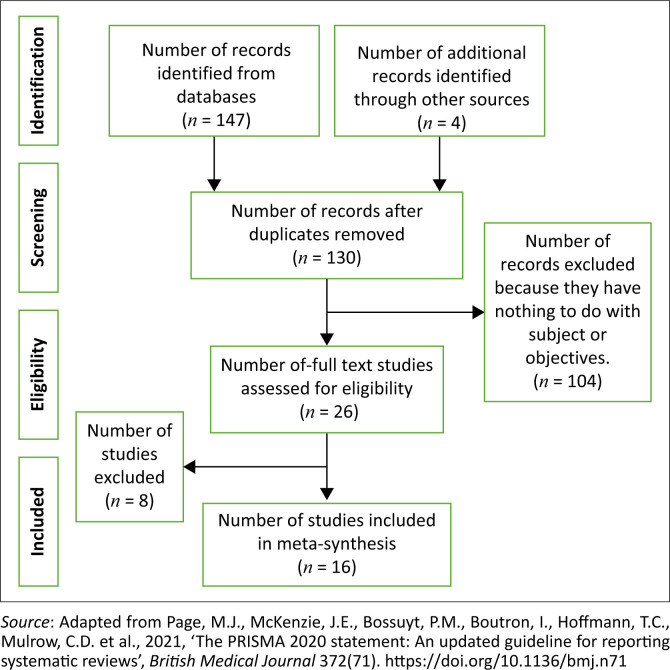
A Preferred Reporting Items for Systematic Reviews and Meta-Analyses (PRISMA) flow diagram of the study selection process.

A total of 147 records were identified from various databases and four additional records were found through other sources after retrospective references. After removing duplicates, the total number of records considered was 130. About 104 records were excluded during the screening process because they were not relevant to the subject or objectives of the study. A total of 26 full-text studies were assessed for eligibility. Eight studies were excluded based on the eligibility criteria. Ultimately, 16 studies were included in the meta-synthesis. Figure 1 shows the Preferred Reporting Items for Systematic Reviews and Meta-Analyses (PRISMA) flow diagram of the process of retrieval and selection of articles for inclusion.

Of the 16 included studies, 14 used qualitative methodologies and two were mixed method studies. The studies were conducted from 2012 to 2024 across five different countries and included 12 from South Africa and one each from Eswatini, Uganda, the United Kingdom and the United States. All studies were published in English. An illustration of the characteristics of included studies with participants, designs, aims, methods of analysis and key findings is presented in a meta-summary (Table 1).

**TABLE 1:** Meta-summary of included studies.

Author(s), (year), country	Aim/purpose	Design and participants	Methods/analysis	Themes/key findings related to ubuntu	Quality appraisal CASP rating score
De Beer and Brysiewicz (2017), South Africa	To explore and describe the conceptualisation of family care during critical illness in KwaZulu-Natal	Grounded theory research method Participants = doctors (*N* = 6) and intensive care nurses (ICNs) (*N* = 16) working in the ICUs of two hospitals (one a private hospital and one public hospital), as well as family members (*N* = 9) of patients admitted to these units in the eThekweni district of KwaZulu-Natal	Data were collected through the use of in-depth individual interviews Data analysis was through open coding and followed by axial coding	Family care is conceptualised as togetherness (ubuntu), partnership (*ukubambisana*), respect and dignity (*Ukuhlonipha*).	10
Magagula (2021), Eswatini	To develop and validate guidelines for maternity care for women with mobility disabilities during pregnancy, labour and puerperium	A qualitative, exploratory, descriptive and contextual research design with a phenomenological approach Participants: (*N* = 12) women with mobility disabilities and (*N* = 12) midwives	Data were collected through in-depth, individual phenomenological interviews and field notes. Purposive sampling was utilised Data analysed through a thematic coding method was applied to analyse the data	Six themes were identified from the data collected from **women** with mobility disabilities: Experience midwives as being cruel and unsupportive Experience of being judged as if it is a sin to be pregnant and disabled at the same time Experience being victimised by midwives Experience of lack of support during pregnancy, labour and puerperium Experience challenges before and after giving birth related to a lack of protocols, equipment and infrastructure Experience the need for support from midwives and family. Three themes identified from the data analysis of the experiences of **midwives:** Physical and emotional efforts required from midwives to provide maternal care to women with mobility disabilities Lack of equipment to meet the needs of women with mobility disabilities Challenges in providing holistic care to women with mobility disabilities during pregnancy, labour and puerperium.	10
Manganyi (2017), South Africa	To develop a short course to inculcate the spirit of ubuntu among the student nurses at Limpopo College of Nursing in Limpopo province	Sequential explanatory research mixed method design Participants: *N* = 115 student nurses for the quantitative phase, and *N* = 10 student nurses for the qualitative phase; and lecturers at a public nursing college	Participants: *N* = 115 student nurses (quantitative phase, utilising self-designed questionnaire) *N* = 10 student nurses (qualitative phase, utilising focus group discussions and reflective diaries). *N* = 10 lecturers utilising focus group discussions Quantitative data analysis was carried out through descriptive analysis Qualitative data analysis was carried out through Tesch’s eight steps	Nursing curriculum lacks ubuntu, mentorship on ubuntu principles is absent for student nurses in clinical areas, and their evaluation does not measure ubuntu understanding in practice. Patient care is influenced by social class, nurses show a lack of sympathy, and socialisation on ubuntu is deficient in nursing training. Incomplete clinical exposure reports, insufficient resources, and lack of assessment by lecturers impede effective ubuntu practice.	10
Matshaka (2022), South Africa	To develop, describe, implement, and evaluate a model as a framework of reference for student nurses to facilitate care through the development of mindfulness	A theory generative, qualitative, exploratory, descriptive, and contextual research design Twenty student nurses	Four focus groups Data were analysed following Saldaňa’s (2013) coding methods	Five themes emerged: (1) The model empowered the participants to love themselves, (2) the model assisted the participants to practise the art of nursing, (3) enabled the environment for model implementation, (4) disenabled environment for model implementation, and (5) suggestions for model implementation.	10
Molala and Downing (2020), South Africa	To gain an understanding of newly qualified critical care nurses’ lived experiences in caring for post-cardiothoracic surgery paediatric patients in a private hospital in Gauteng	Participants were six females with ages ranging from 30 to 34 years, and they were of Black and Indian ethnicity	A qualitative, exploratory, descriptive and contextual research design was employed using Giorgi’s method of data analysis Data were collected employing in-depth, individual phenomenological interviews	Three themes emerged: (1) Participants experienced the Paediatric Intensive Care Unit (PICU) as an overwhelming and stressful environment, (2) participants experienced nursing paediatric cardiac patients to be entirely different from nursing adult cardiac patients, and (3) participants experienced a great need for supervision and training, and a need to be part of the team.	10
Muhammad-Lawal et al. (2023a), South Africa	To explore perceptions of student nurses on the interconnectedness of ubuntu and caring in nursing	A qualitative, exploratory descriptive research design Participants = *N* = 49 fourth-year student nurses enrolled in the undergraduate nursing programme from two universities in the Gauteng province	A focus group interview method, with participants in this focus group limited to no more than eight. Convenience sampling Tesch’s eight-step coding process for thematic analysis	Two themes on perceptions of ubuntu themes emerged: (1) Desire to help and (2) humanness. Two themes on perceptions of caring in nursing emerged: (1) Providing professional help and (2) respect for human dignity. Two themes on perceptions on the interrelatedness of ubuntu and caring in nursing emerged: (1) They are intertwined and (2) dissonance between ubuntu and the practice of caring in nursing.	10
Muhammed-Lawal et al. (2023b), South Africa	To explore the views of the South African final-year student nurses on the ability of ubuntu to foster holistic nursing	Final-year university nursing students	Calaizzi seven steps process of data analysis	Three themes emerged: (1) ubuntu and holistic caring in nursing share common values, (2) ubuntu can be taught and learnt, and (3) ubuntu may not be a sufficient drive for some nurses to provide holistic care.	10
Mulaudzi et al. (2022), South Africa	To explore the perceptions of retired nurses on factors that prevent younger professional nurses from applying the ethos of ubuntu in professional care	Qualitative explorative study Forty retired nurses in Gauteng province	Focus group discussions in a workshop The transcripts were analysed using Braun and Clarke’s (2006) six steps	Two primary themes emerged: (1) Motivation to embrace ubuntu in nursing, and (2) insufficient political will to acknowledge the role of nursing.	10
Nambozi (2014), Uganda	To evaluate a nursing education programme designed to provide practical experience of child health education in two primary schools local to a university in Western Uganda	Explorative qualitative research Pupils, pupil-parent couples, teachers, education administrators, nursing students, faculty staff, university administrators and Florida Atlantic University faculty staff (*N* = 71)	Ubuntu and Western paradigms of participant observation, interviews (focus group, semi-structured interviews, and email interviews), and document analysis An inductive content analysis approach	Seven main categories emerged: (1) Collectivism, being involved, (2) participating, (3) improved communication/reception, (4) acting as a role model, (5) developing/gaining confidence/self-esteem, (6) crossing/bridging the gap, and (7) transforming one’s life.	10
Nash-Patel et al. (2022), United Kingdom	To describe educational storytelling interventions that may help nursing students learn to engage and relate to individuals with intellectual learning disabilities (ILD), their families, and teachers	An ethnographic rights-based evaluation Participants were nursing students in degree and master’s level courses in adult, child, mental health, and learning disability fields registered for the Heritage 2 Health (H2H) Virtual Art and Drama Project course	The StoryAid approach, which was used in the Heritage2Health (H2H) Virtual Art and Drama Project, is highlighted, and the use of storytelling in nursing education using the story *Ubuntu the Lion With the Long Long Mane*	Four themes emerged: (1) Relating to the story and characters, (2) participating in the processes of storytelling, (3) relating to other people participating in the storytelling, and (4) relating shared learning to clinical contexts and professionalism.	10
Ngunyulu et al. (2020), South Africa	To explore and describe the perspectives of nursing students regarding incorporating African traditional indigenous knowledge (ATIK) into the curriculum	A participatory transformative approach Participants comprised nursing students. The academics, traditional health practitioners, indigenous knowledge holders and primary healthcare nurses	Data were collected through one 8-h communal dialogue workshop	Four themes emerged: (1) Politics of identity, (2) displacement and distortion, (3) curriculum content, and (4) institutional resistance.	9.5
Shiluvane (2020), South Africa	To develop a model that could promote moral regeneration among nurses in Limpopo province	A mixed method, multiphase study Participants were different categories of nurses and patients	Questionnaires, semi-structured focus groups and individual interviews	Two themes emerged: (1)Patients’ perceptions of nurses’ ethical-moral behaviours and (2) patients’ views on the ethical-moral behaviour of nurses.	10
Vink and Sefotho (2023), South Africa	To critique incivility as an antithesis of Botho/ubuntu in professional nursing education	Qualitative exploratory and descriptive design Participants: nurse educators (10) and students (15) from a university-based nursing school and a nursing college were purposively sampled for data collection	Data were collected through semi-structured individual, face-to-face interviews	The theme of ‘manifestations of incivility’ emerged from the main categories of disruptive, inappropriate and violent behaviour. The results showed that incivility contradicted the values of care, humanness, compassion and respect that are essential for professional nursing education.	9.5
Msila (2017), South Africa	To investigate the feasibility of incorporating African perspectives into nursing education. Considering the historical colonial roots, the exploration seeks to address the challenge of aligning nursing education with an Africanised framework of education	Qualitative research Predominantly female nurses (*N* = 38) working at three community clinics and three hospitals. Non-probability snowball sampling	Individual open-ended semi-structured in-depth interviews Thematic analysis, incorporating grounded theory elements	The participants had difficulty comprehending and articulating the concept of Africanisation of nursing (ubuntu), and some even questioned its relevance and feasibility. The study also revealed that the participants had no exposure to any African nursing theories or models in their curriculum, while they were familiar with the Western nursing pioneer Florence Nightingale. The study suggested that more research and dialogue are needed to integrate indigenous knowledge and practices into nursing education.	7
Rasweswe et al. (2024), South Africa	To explore the perceptions of nursing students regarding the use of ubuntu in the fight against HIV and TB stigma	Qualitative participatory research An interactive workshop was used to gather data. First- and second-level nursing students enrolled for the R174 degree programme in 2023 academic year at a selected university in South Africa. *N* = 160 nursing students	Critical analyses of data sets	Two themes emerged: (1) Expression of ubuntu in relation to the reduction in HIV and TB Stigma and (2) recognition of ubuntu as a tool to combat stigma associated with HIV and TB.	10
Tyson (2012), United States	This qualitative study explores the critical thinking experiences of African students.	Twelve African nursing students enrolled in several universities in the United States	Semi-structured interview approach Three major frameworks guided the study including, (1) Van Manen’s interpretive hermeneutical approach to qualitative research, (2) the conceptual models of critical thinking described by Sheffer and Rubenfeld’s (2000) nursing consensus statement and (3) Barnett’s (1997) description of criticality and the African concept of ubuntu.	Seven themes emerged: (1) Learning experiences in Africa, (2) using new learning tools to adapt to critical thinking, (3) fear, (4) desire for faculty interaction, (5) cultural factors impeding critical thinking, (6) evolving self-awareness and (7) the voice of those afraid to speak.	10

*Source:* Adapted from Nolte, A.G.W., Downing, C., Temane, A. & Hastings-Tolsma, M., 2017, ‘Compassion fatigue in nurses: A metasynthesis’, *Journal of Clinical Nursing* 26(23–24), 4364–4378. https://doi.org/10.1111/jocn.13766

CASP, Critical Appraisal Skills Programme; TB, tuberculosis

#### Risk of bias (quality) assessment

As this was a synthesis of qualitative studies, a risk of bias assessment was not conducted for the included studies in this synthesis. Instead, quality appraisals were conducted using the CASP checklist. The rating scores for the included studies are displayed in Table 1.

#### Confidence in review findings

The CERQual approach, which guides the assessment of confidence in review findings, was employed in the meta-synthesis (Lewin et al. 2018). Confidence in the Evidence from Reviews of Qualitative research assesses confidence levels based on methodological robustness or limitations, relevance, coherence and data adequacy. High confidence was attributed to studies with minimal methodological issues and strong coherence, relevance and adequacy. In contrast, studies with significant limitations or lacking in coherence and relevance were assigned low confidence. Those with mixed evaluations received moderate confidence. The confidence levels of the reviewed findings are depicted in Table 2.

**TABLE 2:** Confidence profile of evidence from qualitative studies on ubuntu philosophy in nursing.

Theme	Subthemes	Contributing Studies	Methodological limitations	Coherence	Relevance	Adequacy	CerQual confidence	Explanation of confidence in the evidence assessment
Integration of ubuntu principles in nursing education	1. Pedagogical approaches	Manganyi (2017), Nambozi (2014), Nash-Patel et al (2023), Msila (2017), Tyson (2012), Rasweswe et al (2024), Muhammad-Lawal et al (2023a)	Moderate methodological limitations (One study lacked clarity in its data collection aspects, and the researcher did not provide justification for the chosen methods or transparency in data analysis).	No/very minor concerns about coherence	No/very minor concerns about adequacy	Moderate concerns about adequacy	Moderate	Moderate concerns on methodological limitations
	2. Decolonising nursing education	Msila (2017), Ngunyulu et al (2020), Patel et al (2023), Muhammad-Lawal et al (2023a)	One study lacked clarity in its data collection aspects, and the researcher did not provide justification for the chosen methods or transparency in data analysis.	No concerns about coherence	No concerns about relevance	Moderate concerns about adequacy	Moderate	Moderate concerns on methodological limitations
	3. Role-modelling ubuntu	de Beer & Brysiewicz (2016), Mulaudzi et al (2022), Manganyi (2017), Magagula (2021), Molala & Downing (2020), Shiluvane (2021), Muhammad-Lawal et al (2023a), Matshaka (2022), Nambozi (2014), Vink & Monaheng (2023)	No/very minor concerns about methodological limitations	No/very minor concerns about adequacy	No/very minor concerns about adequacy	No/Low concerns about adequacy	High	No concerns regarding methodological limitations

*Source:* Adapted from Lewin, S., Booth, A., Glenton, C., Munthe-Kaas, H., Rashidian, A., Wainwright, M. et al., 2018, ‘Applying GRADE-CERQual to qualitative evidence synthesis findings: Introduction to the series’, *Implementation Science* 13(Suppl 1), 2. https://doi.org/10.1186/s13012-017-0688-3

Note: Overall CERQual rating of confidence in the finding, based on four levels of confidence in the evidence contributing to the finding (Lewin et al 2018): High – it is highly likely that the review finding is a reasonable representation of the phenomenon of interest; Moderate – it is likely that the review finding is a reasonable representation of the phenomenon of interest; Low – it is possible that the review finding is a reasonable representation of the phenomenon of interest; Very low – it is not clear whether the review finding is a reasonable representation of the phenomenon of interest.

### Meta-synthesis analysis

**FIGURE 2:**
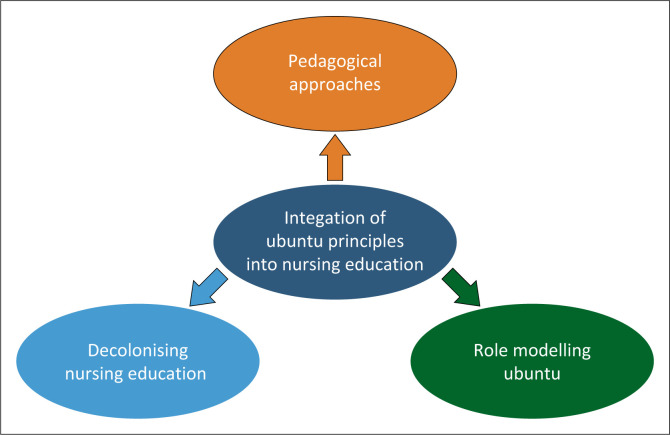
The over-arching theme and subthemes.

The meta-synthesis revealed one meta-theme: *Integration of ubuntu principles into nursing education* and three subthemes: *Pedagogical approaches, decolonising nursing education* and *role modelling ubuntu*. The over-arching meta-theme and its subthemes are displayed in Figure 2.

Nursing in many African countries, much like their healthcare systems, is deeply influenced by biomedical ethical principles and Western values, concepts and theories. Unfortunately, this influence often sidelines the richness and abundance of Africa’s indigenous languages, worldviews, teachings and experiences within the nursing profession (Havenga et al. 2018). As a result, there is a predominance of colonising thinking and actions throughout the nursing profession, including nursing education (Havenga et al. 2018).

To effectively cultivate a culture of ubuntu within nursing education, it is crucial to integrate the principles of this philosophy – compassion, mutual respect and community, deeply into the core of nursing education and interactions. The three emerging subthemes, namely, pedagogical approaches; decolonising nursing education; and role modelling ubuntu, are discussed below.

#### Pedagogical approaches

Ubuntu provides an Afrocentric worldview, promoting interconnectedness among students and educators, and therefore facilitates pedagogic strategies that foster understanding of self and others, building positive relationships, getting students to work together, and creating and developing knowledge together (Chigangaidze et al. 2023; Mugumbate et al. 2023). Ubuntu as a pedagogical approach is rooted in love, respect and dignity, fostering compassionate, harmonious relationships between educators and students. When educators show care, students are inspired to reciprocate in class and clinical practice:

If it was by choice they were going to be at a hotel. We had always been told to treat the patient as if they were all our own family member. (Matshaka 2022:151)

An example of a transformative teaching strategy that was provided in the included literature was storytelling. Through storytelling, students had the opportunity to express their personal truths about preferred ways of creating trust and acceptance of self and others as authentic, fallible, feeling and caring beings (Nash-Patel et al. 2023). Utilising storytelling and oral traditions in teaching can be an effective ubuntu pedagogic strategy. These methods can convey moral lessons, historical narratives and cultural values, making learning more engaging and relatable:

Through storytelling, you are able to open up and facilitate a space for navigating uncertainty. (Nash-Patel et al. 2023:5)

Storytelling encouraged nursing students to ‘deconstruct, reconnect, and rediscover’ their assumptions, and to build understanding in relation to patients such as through exploring no-tions of ability and disability, and what it means to be healthy (Nash-Patel et al. 2023:5).

Integrating community service or community-based projects into the curriculum enables students to apply their learning in real-world contexts. This engagement helps students see the relevance of their studies and the importance of service to others, a value central to ubuntu. Ubuntu-based community nursing education models empower students to learn how to provide nursing care for individuals, regardless of where they encounter them (Nambozi 2014). This communal perspective, rooted in ubuntu principles, highlights the significance of community in nurturing critical thinking during teaching and learning, especially in diverse educational environments. In such diverse educational settings, students engage with each other, influenced by a communal ethos. For instance, students willingly assist peers facing academic challenges, even if it requires personal sacrifice:

We talk freely and exchange ideas … we learnt a lot and when it comes to teaching we find our work much easier as we can explain as well as giving examples. (Nambozi 2014:175)

Central to ubuntu pedagogy is the idea that all learners, regardless of their racial, educational, economic and linguistic backgrounds, as well as sexual orientations, are capable individuals who can excel if their humanity is prioritised in their education (Ngubane & Makua 2021). Teaching and learning grounded in the core principles of ubuntu create a supportive environment that fosters solidarity, cooperation, mutual respect and the sharing of ideas and resources. This approach transforms learning and teaching into a collective, supportive and social process, contrasting with traditional individualistic and competitive educational models. It promotes an equal partnership between teachers and students as co-creators of knowledge. Collaborative learning, a key element of this strategy, emphasises group work and collective problem-solving, encouraging students to work together and embody the ubuntu tenet ‘I am because we are’. This not only enhances knowledge acquisition but also strengthens interpersonal bonds and cultivates a strong sense of community among students:

I guess one difference between the African study group and my American’s friend is with the African we just, our head was everywhere where we could actually talk about it and it would take us hours to come together and make sure the person that didn’t understand understands. Where I study with the Americans it was just kind of like one way and everybody was like on point. (Tyson 2012:151)

Given the significant social and cultural role of nursing education in society, it is crucial for it to emphasise social justice, enabling students to become agents of change and responsible citizens. Teaching social justice from an ubuntu perspective highlights the importance of care, dignity and respect for everyone, regardless of race, ethnicity, gender or sexuality. This pedagogic approach encourages students to engage critically with ethical issues and social justice, focussing on principles of equality, equity and fairness. It aims to advocate for community members or individuals who have been historically marginalised, disadvantaged or excluded economically, politically and socially. Such an educational framework not only broadens students’ perspectives but also empowers them to make impactful contributions to their communities and the broader society:

Social justice is an aspect that needs to be emphasized in the nurses’ training. Many people still struggle to access health care. The role of nurses should go further into ensuring that health care assures equality because the poor people are usually left out when it comes to services such as health. (Msila 2017:8)

#### Decolonising nursing education

Nursing in South Africa has deep colonial roots– a socioeconomic and political system that leveraged the ideology of White supremacy to justify the domination and exploitation of non-White races. This colonial legacy persists, partly because there is an inadequate critical reflection on the Western ethical assumptions, values and principles routinely employed in the nursing profession (Havenga et al. 2018). Compounding this issue, the country’s nursing education system is plagued by long-standing structural and systemic inequalities and continues to prioritise Eurocentric curricula and teaching methods (McGibbon et al. 2014; Ngungulu et al. 2020). Consequently, nurse educators often neglect the ubuntu philosophy, despite its deep roots in African culture and its potential to significantly influence care perceptions. Some individuals still regard indigenous philosophies like ubuntu as inferior to concepts compared to those from other parts of the world, and others lack the confidence to write, discuss, defend, lecture or tutor on the subject (Mugumbate et al. 2023). This colonial influence has led to a significant gap in awareness among some nurses about the value of African traditional indigenous knowledge. This gap is encapsulated in the words of one nurse:

Some patients are stubborn and believe in traditional ways of doing things. (Msila 2017:41)

Ngunyulu et al. (2020) highlighted a significant gap in the historical records, noting the absence of documentation on the contributions of Africans to modern medicine. This oversight in acknowledging indigenous knowledge has roots in historical events marked by oppression and colonisation. The underrepresentation of ubuntu philosophy in educational resources further complicates its integration into academia. Educators often lack access to syllabi, textbooks, journal articles, case studies, codes of ethics, guidelines, models, theories and various digital content that embody ubuntu values (Mugumbate et al. 2023). Addressing this issue requires a deeper examination of our past:

We need to deal with the history of colonisation and slavery. Our history of slavery and colonisation should be the basis for transformation. (Msila 2017:5)

This call to action highlights the urgent need for a transformative approach in education that not only incorporates but also values the rich history and contributions of African knowledge systems. Integrating the ubuntu philosophy into the curriculum is seen as a crucial pathway for decolonisation. This is particularly relevant as the curriculum often acts as a ‘carrier of coloniality’ within educational institutions, according to Le Grange (2021:6). Consequently, failing to challenge the dominant approaches embedded in the curriculum significantly impedes decolonisation efforts:

Ubuntu is one of those philosophies that can be used for the decolonisation of the curriculum. (Manganyi 2017:5)

#### Role modelling ubuntu

This subtheme addresses the process by which new nursing professionals learn the norms, values, behaviours and skills necessary to participate effectively as members of the nursing profession. In the context of ubuntu, this entails the incorporation of these communal and ethical values into daily nursing practices. Role modelling ubuntu involves educators displaying behaviours that align with peace, dialogue and collaboration. As Oviawe (2016:8) notes, role modelling facilitates ‘peaceful co-existence’, a student-centred approach that guides students on how to engage in dialogue, collaborate and self-govern. Educators act as key role models by facilitating and encouraging cooperation and tolerance, and by serving as examples of co-existence through their own behaviour. This approach gives students autonomy, encouraging them to actively participate, collaborate to solve problems and develop a sense of respect, solidarity and equity. Such values align with ubuntu’s emphasis on collective well-being, affirming others’ strengths without feeling threatened by their capabilities. However, there are instances educators display unkindness, harsh treatment or exclusion to students, contradicting the principles of ubuntu, which emphasise empathy, respect and collective well-being:

They eat their young you need to be killed and levelled first before you can be seen as part of the group. So, you get the nurse that is unkind to her young ones. (Vink & Sefotho 2023:21457)

A crucial aspect in integrating ubuntu into nursing education is that educators must not only teach ubuntu values but also live them, creating an environment where respect, empathy and mutual support are part of the everyday learning experience. This can lead to a more compassionate, holistic approach to healthcare, where future nurses are prepared to provide care that honours the dignity and humanity of every patient.

One study (Manganyi 2017) found that as nurse educators practice ubuntu values, they were in actual fact teaching students ubuntu through role modelling, thus rendering ubuntu a pedagogical tool. When educators and senior students role model ubuntu values, it is a reflection of ubuntu’s value of nurturing and supporting others within the community:

Starting from learning area from class. Educators must first be role models to the students. (Manganyi 2017:105)

A nurse born and nurtured in an ubuntu community is socialised through communitarian principles, developing virtues of belonging, commitment and compassion, which emphasise collectivism, solidarity and shared responsibilities (Mulaudzi et al. 2009). An extract supports this assertion:

During clinical accompaniments, lecturers should try to socialise student nurses about this ubuntu. (Manganyi 2017:111)

Role modelling involves experienced nurses demonstrating the principles of ubuntu in their daily interactions with patients and colleagues, serving as live examples for others to follow. Senior nurses and leaders can display behaviours that embody ubuntu, such as expressing genuine care for the well-being of their team, fostering a supportive and inclusive work environment, and participating in community outreach programmes. These actions are instrumental in instilling the values of ubuntu among all staff members.

When nurse educators practice ubuntu in nursing education institutions, student nurses get oriented to professional values, including the ubuntu philosophy, from the start of their training:

Starting from learning area from class. Educators must first be role models to the students. (Manganyi 2017:105)

I mean in any new environment, everyone needs orientation for at least some few weeks until you get to see how things are done, so it is very challenging. (Molala & Downing 2020:5)

Manganyi (2017) emphasises that while role modelling of ubuntu can occur in educational contexts, it is particularly effective in clinical practice. Similarly, in clinical settings, professional nurses who practice ubuntu act as mentors to student nurses. A lack of ubuntu among students reflects poorly on nurse educators, professional nurses and the nursing educational system. Experienced nurses demonstrate ubuntu’s core values in their daily interactions with both patients and colleagues:

Ubuntu cannot be taught theoretically but the best way to teach ubuntu is through role modelling because student nurses learn best what they see from professional staff. (Manganyi 2017:119)

When senior nurses and leaders in health facilities embody ubuntu values, they set a living example for others, promoting a culture of genuine care, inclusivity, tolerance and teamwork within the work environment:

We need effective team in this unit, seniors with knowledge and good interpersonal relationships; and most of the senior nurses can belittle you, make you a failure. (Molala & Downing 2020:5)

By role-modelling ubuntu through the sharing of knowledge, senior professionals demonstrate their passion for the nursing profession and their commitment to passing on wisdom to the next generation. This approach instils in students a profound respect for patients’ lives, grounded in the understanding that their own success is interconnected with the well-being of those they care for, as reflected in the sentiment that ‘they are because of the patients’ (Matshaka 2022:120). As a result, students are empowered to treat patients with the same respect and compassion they would wish to receive themselves.

## Discussion

In this section, we address the central research question: *What insights from qualitative literature on ubuntu philosophy in nursing can be integrated into nursing education to prepare students for the dynamic health system of the 21st century?* The study revealed one meta-theme: Integration of ubuntu principles into nursing education, and three subthemes: Pedagogical approaches, decolonising nursing education and role modelling ubuntu.

### Integration of ubuntu principles into nursing education

This meta-theme emphasises a human-centred, inclusive and collaborative learning environment in nursing. Central to this approach is prioritising the humanity of learners by recognising their diverse backgrounds and treating them with dignity and respect (Ngubane & Makua 2021). Ubuntu fosters a sense of community through collaborative learning, encouraging group work, collective problem-solving and shared achievement, which shifts focus from individual competition to cooperation (Hlatshwayo, Shawa & Nxumalo 2020).

Embedding values such as empathy, care and compassion helps students connect with others’ experiences, promoting patient-centred care. Additionally, ubuntu highlights social justice, preparing students to advocate for marginalised groups and become responsible citizens who value equality and fairness (Ngubane & Makua 2021). Thus, curricula should enable real-world application of knowledge, reinforcing the connection between theory and practice and the value of serving others (Nambozi 2014).

Inclusivity and diversity are fundamental to ubuntu, encouraging respect for different cultural, linguistic and social backgrounds. Therefore, the educational institution should provide a supportive learning environment, where every student feels valued and empowered irrespective of their cultural, linguistic and social backgrounds. Ubuntu promotes shared responsibility, ethical engagement and mutual respect, creating a culture that values collaboration and community. This can be accomplished by embedding ubuntu principles, namely, respect, dignity, compassion, solidarity and survival into course design, content, teaching and assessments (Hlatshwayo et al. 2020). Thus, principles of understanding the self and others, building positive relationships, nurturing students’ minds, encouraging teamwork and collaboration, teaching with love and care, and leveraging students’ linguistic resources should be embodied in all teaching and learning aspects of nursing education (Ngubane & Makua 2021).

#### Pedagogical approaches

An essential component in integrating ubuntu philosophy into nursing education is the adoption of a humanising pedagogy that is deeply rooted in students’ cultural experiences (Ukpokodu 2016). Ubuntu pedagogy offers a framework that emphasises viewing students as valuable human beings deserving of dignity and respect throughout the educational process. This approach seeks to restore students’ humanity by fostering an environment of genuine love and care in teaching. Such graduates are likely to value the humanity of others, an essential quality for effective and compassionate nursing practice.

Nurse educators play a crucial role in creating empowering learning environments that promote participatory democracy, mutual respect and a deep love for humanity (Chimbi & Jita 2022). By cultivating spaces where both educators and students can freely learn and co-learn, they foster power relations that prioritise humanism, respect and shared responsibility. In this context, democracy is not just a theoretical concept but a lived experience, with educators acting as facilitators while students gain autonomy and ownership of their learning journey (Ukpokodu 2016). This suggests that teaching methods should adopt humanising instructional strategies, such as cooperative learning, where students engage in partnerships, small groups and teams, benefiting from mutual, individual and collective learning (Ukpokodu 2016).

To reflect the communal aspects of ubuntu, teaching practices may include collaborative and participatory learning activities (Dison & Collett 2023). Furthermore, engaging and culturally resonant methods such as storytelling, oral narratives, folklore, cultural artefacts, music, dialogue and debate (Waghid 2013) can be used. By incorporating these elements, students and educators can share their traditions and practices, making the learning experience more relatable, authentic and impactful. Such practices not only enhance academic empowerment but also help cultivate personal success, empathy and a sense of community, all of which are vital in nursing education.

#### Decolonising nursing education

The systemic disregard for indigenous knowledge has led to a significant gap in the historical narrative, omitting the contributions of Africans to modern healthcare practices (Ngunyulu et al. 2020). Such omissions are deeply rooted in a history of colonisation, where local knowledge was devalued, marginalised or excluded.

To address these issues, there is a pressing need for transformative approaches that actively incorporate and celebrate the contributions of African knowledge systems within nursing education. Integrating ubuntu into the curriculum is essential for decolonisation, as the curriculum often acts as a ‘carrier of coloniality’, maintaining outdated, exclusionary worldviews that undermine the richness of local traditions (Le Grange 2021:5). By challenging these dominant approaches, educators can foster a more inclusive and contextually relevant education system.

Nurse educators play a pivotal role in this transformation. Drawing on Murrell Jr.’s (2000:340) concept of ‘community teachers’, educators should cultivate the cultural competencies needed to recognise and address systemic inequities and dehumanisation. These educators, empowered by their cultural and community knowledge, can advocate for social justice, support underserved communities and advance the decolonisation agenda (Matahela 2019; Matahela & Van Rensburg 2023). This transformation calls for a comprehensive review of current curricula to eliminate Western epistemological dominance and integrate diverse worldviews that honour the dignity and history of formerly colonised people (Heleta 2016).

Practical steps for integrating ubuntu into nursing education include revising teaching materials to reflect diverse cultural perspectives, fostering partnerships with local communities to enrich students’ understanding and equipping educators with the necessary tools to confidently engage with indigenous philosophies. By valuing and embedding ubuntu principles, nursing education can progress towards a decolonised, humane and inclusive approach that better reflects the cultural contexts of African communities.

#### Role modelling ubuntu

Role modelling ubuntu in nursing education is crucial for fostering a culture of care, respect and inclusivity. When senior nurses and health care leaders embody ubuntu values, they set a living example, cultivating a work environment rooted in genuine care and collective well-being. This aligns with ubuntu’s emphasis on the intrinsic worth of every individual, teaching nurses that their purpose is interconnected with their patients’ well-being (Matshaka 2022).

Role modelling is impactful in both educational and clinical settings. As Manganyi (2017) notes, professional nurses who practice ubuntu serve as mentors, guiding students in adopting values such as empathy, respect and collaboration. The absence of these values in students can reflect a lack of consistent role modelling by educators, emphasising the need for ubuntu to be integrated across all levels of nursing.

Educators who embody ubuntu introduce students to professional norms and skills through role modelling, facilitating cooperation, dialogue and self-governance (Oviawe 2016). However, behaviours that contradict ubuntu, such as harshness or exclusion, undermine these values, highlighting the need for consistent practice. Addressing this requires nurturing ubuntu culture through socialisation, role modelling and orientation. Senior nurses can demonstrate empathy and respect, guiding young nurses to adopt these principles, thus fostering a commitment to care and unity that strengthens the nursing profession.

Ubuntu principles can be embedded in the curriculum, but they may also be transmitted through a hidden curriculum, especially during orientation processes and daily activities (Nkondo 2007). Educational institutions can inculcate these principles by integrating them into institutional programmes, including the orientation period, classroom instruction and clinical practice. Furthermore, institutions should align their vision, mission and values with ubuntu principles, ensuring a consistent and clear application of these values in personnel decisions. While educators model ubuntu during teaching and learning activities, managers should also ‘walk the ubuntu walk’, embodying these principles in their daily management practices, thereby fostering a culture of respect, compassion and collective responsibility.

### Recommendations

Based on the study’s findings, it is recommended that nursing education revise curricula to incorporate ubuntu principles while encouraging educators to model these values. For nursing practice, mentorship programmes should be established where senior nurses guide new graduates in embodying ubuntu values, supported by ongoing professional development. Lastly, nursing research should focus on generating empirical evidence of the impact of ubuntu on nursing practice and patient outcomes, alongside the development of frameworks to support its integration into nursing education.

### Strengths and limitations

Strict inclusion criteria ensured that only studies on ubuntu in nursing with implications for nursing education were considered. The meta-synthesis was conducted by investigators within the same field, representing the most common approach. One potential weakness is that, as a meta-synthesis, the study involved synthesising studies conducted by researchers different from those who carried out the original research. This could have led to varied interpretations. In addition, as with many systematic reviews, quality appraisal and confidence assessment efforts for this meta-synthesis were not conducted to exclude studies but to assist authors in determining which studies were more dependable in terms of evidence and methodological rigour. The confidence scores assessed the quality and relevance of studies on ubuntu philosophy in nursing education, ensuring only those with a strong representation of key insights were included. A potential limitation, however, is that this approach may exclude studies with useful insights because of lower scores from methodological differences, potentially affecting the comprehensiveness of the meta-synthesis.

Lastly, studies were predominantly from South Africa, which may limit the case-to-case transferability of findings to other contexts. However, analytical generalisation to similar contexts could be valuable in informing future research.

## Conclusion

This meta-synthesis sought to identify key aspects of the ubuntu philosophy that can be integrated into nursing education by analysing studies that explore its key components in nursing. The identified elements to be integrated and described through this study’s themes, namely pedagogical approaches, decolonising nursing education and role modelling. Ubuntu could transform nurse training to better meet community needs, fostering ethical, compassionate and culturally sensitive nurses. As South Africa continues to address the legacies of colonialism and racial divisions, the demand for culturally competent healthcare is imperative. The researchers hope this study contributes to the ongoing discourse on integrating indigenous philosophies like ubuntu into nursing education and aligning nursing care with local cultural and ethical values to promote more effective and humane practices.
